# Reduced habituation of auditory evoked potentials indicate cortical hyper-excitability in Fragile X Syndrome

**DOI:** 10.1038/tp.2016.48

**Published:** 2016-04-19

**Authors:** L E Ethridge, S P White, M W Mosconi, J Wang, M J Byerly, J A Sweeney

**Affiliations:** 1 Department of Pediatrics, Section on Developmental and Behavioral Pediatrics, University of Oklahoma Health Sciences Center, Oklahoma City, OK, USA; 2 Department of Psychology, University of Oklahoma, Norman, OK, USA; 3 Department of Psychiatry, Center for Autism and Developmental Disabilities, University of Texas Southwestern Medical Center, Dallas, TX, USA; 4 Department of Pediatrics, University of Texas Southwestern Medical Center, Dallas, TX, USA; 5 Departments of Applied Behavioral Science and Psychology, Schiefelbusch Institute for Life Span Studies and Clinical Child Psychology Program, University of Kansas, Lawrence, KS, USA

## Abstract

Sensory hypersensitivities are common, clinically distressing features of Fragile X Syndrome (FXS). Preclinical evidence suggests this abnormality may result from synaptic hyper-excitability in sensory systems. This model predicts reduced sensory habituation to repeated stimulus presentation. Fourteen adolescents and adults with FXS and 15 age-matched controls participated in a modified auditory gating task using trains of 4 identical tones during dense array electroencephalography (EEG). Event-related potential and single trial time–frequency analyses revealed decreased habituation of the N1 event-related potential response in FXS, and increased gamma power coupled with decreases in gamma phase-locking during the early-stimulus registration period. EEG abnormalities in FXS were associated with parent reports of heightened sensory sensitivities and social communication deficits. Reduced habituation and altered gamma power and phase-locking to auditory cues demonstrated here in FXS patients parallels preclinical findings with *Fmr1* KO mice. Thus, the EEG abnormalities seen in FXS patients support the model of neocortical hyper-excitability in FXS, and may provide useful translational biomarkers for evaluating novel treatment strategies targeting its neural substrate.

## Introduction

Fragile X Syndrome (FXS) is a neurodevelopmental disorder associated with intellectual disability, facial dysmorphology and social deficits such as extreme shyness and anxiety.^1^ Auditory hypersensitivity is particularly common.^2, 3, 4^ While animal models for sensory hypersensitivity have been developed, little is known about neural underpinnings of this abnormality in FXS patients.

Animal models of FXS point to neuronal network hyper-excitability as a possible cause of sensory hypersensitivity. Local circuit hyper-excitability in *Fmr1* knockout mouse models of FXS has been demonstrated, including prolonged ‘UP' states in the gamma frequency range, decreased glutamatergic drive on fast-spiking GABAergic inhibitory neurons in sensory cortex^5^ and heightened neurophysiological response to auditory stimuli.^6^ Inhibitory circuit deficits in these mouse models have been linked to abnormalities in both fast-spiking parvalbumin-expressing inhibitory interneurons^5, 7^ and somatostatin-expressing low-threshold-spiking inhibitory interneurons;^8^ these alterations may contribute directly to local circuit disorganization and hyper-excitability. Disorganized and hyper-excitable networks can have increased cortical ‘noise' or desynchronous firing in the gamma frequency range that reduces stimulus selectivity and lowers the impact of local network output on postsynaptic circuits.^9^

Electroencephalography (EEG) can be used to characterize auditory processing abnormalities and cortical hyper-excitability in FXS. To date, few EEG studies in FXS have been conducted, especially studies of evoked sensory responses.^10^ The most common finding in FXS EEG studies has been enhanced early-sensory (N1) event-related potential (ERP) responses to auditory stimuli, which is consistent with neocortical hyper-excitability models of FXS.^11, 12, 13^

One approach for using EEG to assess sensory hypersensitivity is to utilize gating-type paradigms to determine whether there is a reduced N1 response to repeated stimulus presentations, reflecting reduced neural habituation in sensory cortex. The mechanism for sensory or short-term N1 habituation is believed to be related to refractoriness of cell populations involved in basic sensory processing systems rather than more complex cognitive processes exerting top–down control of sensory cortex.^14, 15^ In typically developing subjects, reduced N1 suppression during sensory gating tasks have been associated with increased attention to irrelevant sounds^16^ and sensory avoidance on the Adolescent and Adult Sensory Profile,^17^ suggesting a link between N1 habituation and auditory hypersensitivity.

There is preliminary EEG evidence that individuals with FXS do not habituate to repeated sounds.^11, 18^ However, previous habituation studies in FXS utilized an auditory oddball paradigm, a task in which rare novel (oddball) tones are presented in the context of a majority of standard (repeated) tones. Adding this probe of expectancy effects adds cognitive complexity and variable attentional influences to the assessment of basic auditory processing.^12, 16, 19, 20, 21^ Second, stimuli in prior studies were separated by relatively long inter-stimulus intervals, which can diminish habituation effects, as refractory circuits significantly complete their recovery after intervals longer than 500 ms.^22, 23^ When tones are presented in sequence with a shortened inter-stimulus interval and without novel distracters, cognitive factors are limited and habituation effects seen in N1 reduction to repeated tones are increased. Limiting cognitive factors is particularly important when assessing sensory processing in groups with cognitive deficits such as FXS, where group differences in cognitive processing could obscure results. Thus, habituation might be better assessed using shorter inter-stimulus intervals (500 ms ISI) in a passive listening paradigm.

Time–frequency analyses have revealed a relationship between increased power in the gamma frequency range and N1 amplitude abnormalities during sensory gating for other disorders with habituation deficits. These have been related to regulation of inhibitory interneuron circuits in sensory cortex, which act to bind stimulus features.^24^ Time–frequency analyses of evoked EEG data to our knowledge have not been conducted in FXS, but may be useful to investigate neural correlates of local circuit hyper-excitability, particularly in the gamma range where deficits have been observed in *Fmr1* mice and proposed to underlie sensory processing abnormalities.^5^

The present study investigated ERP and time–frequency components of short-term habituation to repeated tones in auditory cortex in individuals with FXS and healthy matched controls. Gamma activity was investigated to determine if there were similar increases in power in this frequency band in response to tones as has been reported in somatosensory cortex in *Fmr1* mouse slice preparations,^5^ and if alterations in gamma activity were related to abnormalities in N1 habituation deficits as might be expected due to the role of gamma oscillations in maintenance of synchronous population activity in local cortical circuits. Electrophysiological abnormalities were correlated with parental reports of sensory and behavioral deficits in FXS patients to evaluate their relationship to sensory hypersensitivities and other behavioral problems associated with FXS.

## Materials and methods

### Participants

Fourteen adolescents and adults with full mutation FXS (mean age=28.5, s.d=11.7; age range 14–57; 3 females) and 15 age-matched controls (mean age=28.9, s.d=10.2; age range 16–55; 5 females) participated in the study. Previous studies support this sample size as sufficient to detect group differences.^11, 12, 13, 18^ Healthy controls had no known prior diagnosis or treatment for psychiatric illness. Exclusion criteria included history of seizures and current use of anticonvulsant medications including benzodiazepines, or novel potential treatments for FXS (minocycline). Four patients were receiving atypical antipsychotics, and three antidepressants, all on a stable dose for at least 4 weeks. Medicated patients did not differ on primary EEG measures.

Clinical questionnaires including the Adolescent and Adult Sensory Profile,^25^ Social and Communication Questionnaire (SCQ), Achenbach Adult and Child Behavior Checklists (ABCL), and Aberrant Behavior Checklist (ABC) were completed for FXS participants by their primary caregiver. Intelligence quotient (IQ) of FXS participants was assessed with the Stanford–Binet Intelligence Scale 5th Edn,^26^ which characterizes intellectual ability across a broad ability range. IQ of controls was estimated using the briefer Wechsler Abbreviated Scale of Intelligence (WASI).^27^ All participants provided written informed consent (caregiver with assent or individual consent as appropriate) prior to participation, as approved by the UT Southwestern Institutional Review Board.

### Procedure

Stimuli consisted of 150 trials; each trial consisted of a train of four 1000 Hz tones (50-ms duration). Tones within each train were separated by a 500-ms inter-stimulus interval; trials were separated by a 4000-ms interval. Tones were delivered at 65 db through headphones, while participants underwent dense array EEG. Participants watched a silent movie to facilitate compliance.

### ERP recording

EEG was continuously recorded and digitized at 512 Hz, with a 5th-order Bessel anti-aliasing filter at 200 Hz, using a 128 channel BioSemi ActiveTwo system (BioSemi, Amsterdam, Netherlands) with sensors placed according to the International 10/10 system.^28^ All sensors were referenced to a monopolar reference feedback loop connecting a driven passive sensor and a common mode sense active sensor, both located on posterior scalp.

### EEG analysis

Raw data were visually inspected offline. Bad sensors were interpolated using spherical spline interpolation implemented in BESA 6.0 (MEGIS Software, Grafelfing, Germany). Data were digitally filtered from 0.5 to 55 Hz (6 db and 12 db per octave rolloff, respectively; zero-phase) and re-referenced to average reference. Eye movement, cardiac and muscle movement (EMG) artifacts were removed blind to group using independent components analysis (ICA) implemented in EEGLAB 11 (ref. 29) in Matlab (The Mathworks, Natick, MA, USA). Data were epoched into 3000 ms trials (−500 to 2500 ms), averaged across trials and baseline-corrected using the 500-ms period prior to the first tone in each trial. Any trial with post-ICA amplitude exceeding 100 μV was considered residual artifact and removed prior to averaging. All subjects retained at least 75% of trials in the final analyses (FXS *M*=137.6, s.d.=8.8; Control *M*=144.3, s.d.=4.3).

To take advantage of the dense electrode array and integrate data from every sensor, spatial principal components analysis (PCA) was implemented on the grand average ERP.^30, 31^ Component weights were multiplied by each subject's average data, summed across sensors and divided by the sum of the component weights, reducing waveforms from one for each sensor to one waveform per component with a defined spatial distribution across the scalp.

Spectral analyses using Morlet wavelets with 1 Hz resolution were conducted on epoched single trial data weighted by the average PCA component topographies. To balance time resolution in lower frequencies with stability in higher frequencies,^32^ wavelets were calculated using a linearly increasing cycle length from 1 cycle at the lowest frequency (2 Hz) to 10 cycles at the highest (55 Hz). Single trial power (STP) and inter-trial coherence (ITC) measures were obtained to evaluate amplitude of response at each frequency and how stable or phase-locked responses were across trials, respectively.^29^ STP and ITC values were averaged over trials for each individual and transformed into time–frequency plots downsampled to 300 time-bins.^33^ Group differences were calculated using two-sided *t*-tests at each point in the time–frequency matrix. In particular, based on mouse model studies^5, 7, 8^ we predicted that FXS participants would show non-stimulus-specific increases in gamma STP due to hyper-excitable or ‘noisy' local cortical networks.

Control for multiple comparisons was achieved using time–frequency clustering techniques and Monte Carlo simulations.^30, 33^ To maintain a family-wise α of 0.01, a minimum of three sequential time-bins and three adjacent frequencies were required to be significant at a nominal threshold of *P*<0.05.

### Statistical analysis

Amplitude and latency of the N1 component to each tone was measured for each individual at the peak, defined as the most negative-going waveform deflection between 50 and 150 ms post stimulus, and verified through visual inspection.

We limited the number of repetitions to 3 as prior habituation literature suggests that the majority of habituation effects are present by that time.^34^ The exponential decay function is not well-suited to datasets with such a limited number of time points. Therefore habituation of N1 amplitude across repeated tones in a trial was quantified as percent change from the first tone in each trial, with a focus on comparing fast decay (habituation to the first repeated tone) with asymptote (habituation to the fourth tone) to quantify habituation effects. Percent change across repetitions was analyzed using a repeated measures analysis of variance comparing repetition number in sequence (first, second or third) by group (FXS vs control).^34^ All repeated measures tests included Greenhouse–Geisser correction. Latency and amplitude were measured for the P2, defined as the largest positive-going peak between 150 and 250 ms post stimulus. The N2 component was less easily visualized in some participants, so it was quantified as the average amplitude over 30 ms centered on the N2 peak amplitude in the grand average and latency was not calculated.

Clinical correlations were examined with N1 amplitude to the initial tone, average N2 amplitude across all four tones, percent change in N1 amplitude, gamma STP and all significant time–frequency clusters using Spearman's *ρ*. To examine whether such an abnormal increase in high frequency ‘noise' was associated with abnormalities in habituation, correlations between gamma STP, gamma ITC, low frequency ITC, N1 amplitude and N1 percent change were conducted using Spearman's *ρ*. Clinical scales were prioritized based on their applicability to sensory and social abnormalities (Sensory Profile, SCQ). Clinical correlations are presented as exploratory, not corrected for multiple comparisons.

## Results

### Clinical and demographic

FXS patients showed significantly lower full scale IQ than controls (Table 1). Most of the patients scored in the intellectually disabled range, with three patients scoring in the low-normal range; however, EEG and clinical values did not differ for these patients, so they were retained in the analyses. FXS showed significantly higher Sensory Profile and SCQ scores than controls (Table 1).

### Event-related potential

**Figure 1:**
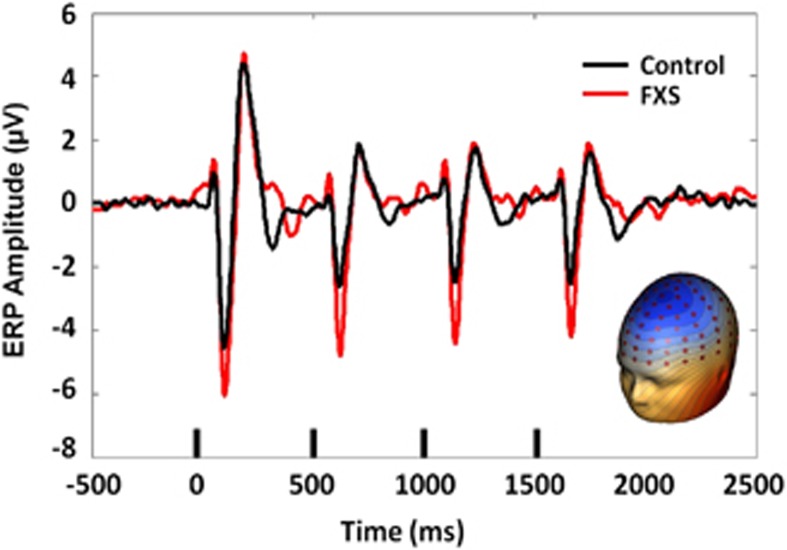
ERP grand average PCA-weighted virtual channel plot for FXS and matched controls, with inset PCA spatial component topography. Small black bars indicate presentation of the auditory stimulus. ERP, event-related potential; FXS, Fragile X Syndrome; PCA, principal components analysis.

Spatial PCA revealed a single spatial component representing 93.0% of the variance that was consistent with known auditory N1/P2/N2 topography (Figure 1). Subsequent analyses were performed on a virtual sensor created by weighting ERP or trial-wise EEG data by the PCA component weights. There was no significant group difference in N1 amplitude to the initial stimulus, *t*(27)=1.7, *P*=0.09. In a repeated measures analysis of variance on percent change across repetitions, there was a main effect of group, F(1,27)=6.9, *P*=0.014, indicating that FXS showed less habituation of the N1 ERP than healthy controls (Figure 1). There was also a main effect of repetition number, F(2,54)=4.7, *P*=0.016. Subjects showed a significant increase in habituation between repetition 1 and 2, *t*(27)=2.54, *P*=0.17, (mean percent change repetition 1=31%, s.d.=21% mean percent change repetition 2=38%, s.d.=18%) but no difference in habituation between repetitions 2 and 3, indicating asymptote of habituation by the fourth stimulus in the trial, consistent with findings in typically developing individuals.^34, 35^ The repetition number by group interaction was not significant, indicating no difference in rate of habituation between groups. This result suggests that additional repetitions do not result in eventual normalization of N1 habituation by allowing FXS to ‘catch up' with more gradual habituation of sensory responses. Gender was entered as a covariate but was not significant, so was removed from the final model. There was no group difference in N1 latency or P2 amplitude or latency for any of the four stimuli.

N2 amplitude did not show a significant habituation effect, so amplitude was collapsed over all four stimuli. There was a significant group difference in overall N2 amplitude, *t*(27)=3.7, *P*=0.001, with FXS participants showing significantly reduced N2 amplitude (mean=0.19 μV, s.d.=0.73) relative to healthy controls (mean=−0.88 μV, s.d.=0.82).

### Time–frequency analyses

**Figure 2:**
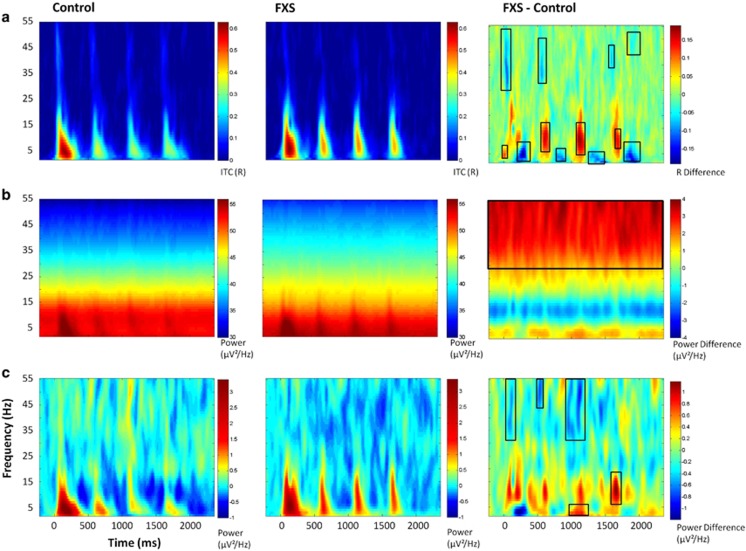
(**a**) ITC. (**b**) Single trial power. (**c**) Baseline-corrected single trial power. Black boxes in the difference maps indicate clusters with significant group differences. Warmer colors in the difference maps (right column) indicate higher phase-locking or higher power for FXS and cooler colors indicate higher values for healthy controls. FXS, Fragile X Syndrome; ITC, inter-trial coherence.

Because baseline STP differed between groups, with FXS showing increased baseline gamma power compared with controls (*t*(27)=3.36, *P*=0.002; Figure 2b), STP was analyzed both as absolute power with no baseline-correction to show overall power differences between groups and also using the pre-stimulus period from −220 to −50 ms (chosen to avoid windowing effects) to baseline-correct STP for each individual to examine relative STP changes associated with stimulus-related neural activity. Point-by-point *t*-tests on time–frequency plots for ITC, STP and baseline-corrected STP (corrected for multiple comparisons) revealed 18 time–frequency clusters with significant differences between FXS and controls (Figure 2, Table 2). Cluster names are identified by stimulus number (initial stimulus or repetition 1, 2 or 3), ERP associated with the time period (if applicable) and frequency band. Time ranges are given relative to initial stimulus onset, to provide equivalency with the *x*-axes in Figure 2. Cluster peaks were identified for highest *t*-values in group comparisons, not for peaks of activity. For absolute gamma STP, a peak statistic is reported, however, group differences were remarkably stable throughout the trial. In general, FXS patients showed significantly increased phase-locking in low frequencies during the N1 time period and decreased phase-locking during the N2 time period (Figure 2a), consistent with the increased amplitude of the N1 ERP and decreased amplitude of the N2 ERP. FXS showed decreased gamma phase-locking during the N1 time period (Figure 2a), and a general increase in absolute gamma power throughout the trial (Figure 2b).

Due to significant group differences in low frequency ITC clusters for each repetition, which paralleled N1 ERP findings, identical habituation analyses were performed for ITC using cluster means. Because a group difference cluster of similar size did not exist for low frequency ITC to the initial stimulus presentation, ITC data for each subject was averaged over a representative time period of 0–150 ms post stimulus using the same low frequency range in which group differences were found for the repetitions. Similar to the ERP results, FXS showed significantly less percent change in low frequency ITC relative to controls F(1,27)=13. 23, *P*=0.001. There was no effect of repetition number or group by repetition interaction.

### Clinical correlations

Significant correlations with clinical measures in FXS participants are presented in Table 3. Increased N1 amplitude to the initial stimulus and reduced habituation were related to clinical measures of sensory and behavioral reactivity.

### Gamma power and ERPs

For FXS, increased gamma STP above the elevated baseline to the initial stimulus was correlated with less habituation to the first repetition (*ρ*=−0.61, *P*=0.02), and increased gamma STP to the first repetition was correlated with less habituation to the second repetition (*ρ*=−0.57, *P*=0.03). Absolute increases in gamma STP throughout the trial were significantly correlated with decreases in gamma ITC to the initial stimulus (*ρ*=−0.54, *P*=0.04), which in turn was correlated with increased N1 ERP to the same stimulus (*ρ*=−0.59, *P*=0.03). Increased gamma power in FXS, both absolute increases relative to healthy controls and further increases above an already elevated baseline, can be thought of as an increase in background neural ‘noise'. This increase in non-specific high frequency neural activity contributed to a decreased ability to synchronize gamma frequency activity when necessary (during initial stimulus representation), which was associated with an overgeneralized excitatory response (increased N1 amplitude), as well as lowered ability to habituate to repetition.

**Figure 3:**
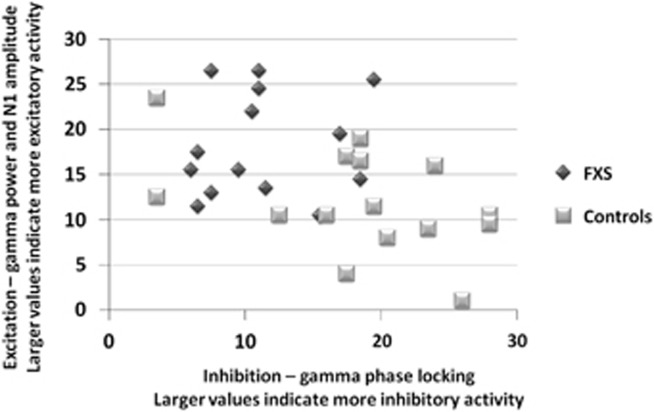
Relationship of excitatory (gamma single trial power, N1 amplitude to initial stimulus) and inhibitory (gamma phase-locking during ‘gamma spike' for initial stimulus and first repetition) activity in response to trains of auditory stimuli. Units for both *x* and *y* axes represent averaged rank scores from lowest (1) to highest (29) for the composite variables. Controls show high inhibitory, organized activity and low background excitatory noise. In contrast, most FXS participants have both higher excitatory response and lower inhibitory activity. FXS, Fragile X Syndrome.

To obtain an overall view of excitatory vs inhibitory abnormalities in FXS, all subjects were ranked from 1–29 in order from lowest to highest individual value for the primary variables indicating change in excitatory (gamma STP, N1 amplitude to initial stimulus) and inhibitory (gamma phase-locking during ‘gamma spike' for initial stimulus and first repetition) activity, then composite variables were created by averaging these rankings in each category. Composite variables were plotted for FXS and control participants to characterize the relative imbalance in excitatory vs inhibitory activity (Figure 3). Controls show high inhibitory, organized activity and low background excitatory noise. In contrast, most FXS participants have both higher excitatory response and lower inhibitory activity.

## Discussion

Sensory processing abnormalities are common and clinically distressing features of FXS. Inability to habituate to sensory events and ongoing background noise may contribute to hypersensitivity to sounds and overstimulation. Behavioral and physiological findings have suggested deficits in short-term habituation in FXS, including reduced prepulse inhibition of the startle response^36, 37, 38^ and abnormalities in electrodermal activity to repetitive stimuli,^39^ however, neural correlates for these findings have not been elucidated. We believe the current study provides the first direct clinical evidence for a contribution of gamma band abnormalities, suggesting an imbalance of excitatory vs inhibitory activity, an effect found in mouse models of FXS, and the first evidence that these are clinically relevant by virtue of their relationship to parental reports of sensory sensitivities in FXS patients.

The current study also provides the first direct evidence of short-term local circuit-based habituation deficits in FXS patients. FXS patients showed a significant decrease in N1 suppression (both ERP amplitude and low frequency phase-locking) to repeated stimuli. This alteration was also associated with parental reports of auditory hypersensitivity and social problems in FXS participants. N1 habituation did not normalize across additional repetitions of the stimulus, suggesting a fundamental deficit in organization of local networks representing basic auditory stimulus properties. Although overall N1 amplitude has been the focus of previous work,^11, 12, 13^ N1 amplitude to the initial non-habituated stimulus did not differ between groups (though there was a non-significant trend) and it was not significantly correlated with habituation (*ρ*=0.15, *P*=0.61), suggesting potentially dissociable mechanisms between neural excitation (initial N1 amplitude)^10, 11^ and inhibition (habituation).^14^

While N1 amplitude to the initial non-habituated stimulus did not differ between groups as in some previous studies,^11, 18^ increased N1 amplitude and increased low frequency ITC during the N1 time period that contributes to formation of the N1 ERP,^40^ were correlated with increased abnormalities on the Sensory Profile, suggesting that individuals with high ERP amplitudes may have more severe sensory symptoms. Previous studies finding N1 differences used younger^11, 18^ and/or smaller^11^ samples, and their level of sensory sensitivities is unknown. The current finding of decreased N2 amplitude in FXS is consistent with findings of some^11^ but not all prior studies.^13^ The long inter-stimulus interval single stimulus task employed by Knoth *et al.*^13^ may have decreased N2 amplitudes and their utility for detecting deficits. The N2 ERP is commonly associated with incorporation of frontal generators in stimulus identification and maintenance of auditory memory traces.^41^ Decreased amplitude of N2 was associated with increased scores on the SCQ, Sensory Profile and ABC Irritability component, suggesting a broad sensory and behavioral impact of this alteration.

Using time–frequency analyses of evoked EEG for the first time in FXS, the current study provides evidence for both increased gamma ‘noise' (increased desynchronous high frequency firing) and decreases in ability to organize inhibitory networks to synchronize gamma band activity in the brain. Broad increases in excitation of gamma circuits without corresponding increases in synchronization of those circuits are consistent with presence of heightened neocortical excitability in FXS, and may represent a mechanism underlying sensory hypersensitivities in this disorder and the observed neural habituation deficit.

Higher ongoing or background gamma power in FXS relative to controls was not locked in time to stimulation. Individuals with elevated background gamma activity showed decreased ability to coherently organize and phase-lock gamma activity during the ‘gamma spike' period, a short burst of phase-locked gamma activity that normally occurs during early-stimulus processing.^42^ Decreased phase-locking during the gamma spike was associated with greater N1 amplitudes, suggesting an overall increase in neural background noise that contributes to both hyper-excitability (increased N1) and disorganization (decreased ability to ‘lock in' or synchronize gamma response to the stimulus). Transient increases in gamma power above the already elevated baseline in response to each stimulus were associated with poorer habituation response to the following stimulus, suggesting that this increase in power is not beneficial or compensatory, but rather reflects an over-recruitment of local networks, which generally oscillate in the gamma range.^43^

In a normally functioning local circuit, refractory properties of the network prevent the same circuit from activating as strongly to repeated stimuli presented in close succession, creating short-term habituation.^14^ Less synchronized and more widely excitable local synaptic networks would mean that the same circuit of synapses may not be excited in the same way for each repetition, preventing proper functioning of the refractory system. Long-term depression, which weakens synaptic connections in a neural circuit, is enhanced in *Fmr1* knockout mouse models of FXS;^44^ weaker connections between neurons in a given local circuit coupled with increased firing of unrelated neurons may also account for that circuit's inability to reliably activate and thus habituate to a repeated stimulus. Importantly, these findings suggest not only a promising avenue for investigating brain dysfunction in FXS, but a promising translational strategy for integrating rodent and patient studies and developing mechanistically important biomarkers useful for advancing drug discovery.

Gamma hyper-excitability in *Fmr1* knockout mice has been related to decreased excitatory drive on fast-spiking inhibitory interneurons, which is associated with decreased synchronization or phase-locking in the gamma range.^5^ Decreased activation of fast-spiking inhibitory interneurons that operate in the gamma range and drive the local neural oscillatory response could account for both local circuit hyper-excitability and reduced neural habituation. Reduced inhibitory drive onto pyramidal cells in auditory cortex from fast-spiking interneurons would lead to increased cell firing and less coherent organization and tuning to sensory stimuli. In the current study, increases in non-phase-locked gamma power were correlated with decreases in phase-locked gamma activity, suggesting a hyper-excitable but disorganized system consistent with these circuit dynamics.

The decrease in phase-locked gamma was also correlated with increased N1 amplitudes, suggesting a connection between local circuit hyper-excitability and increased recruitment of neural generators for sensory evoked potentials. Decreased habituation to a given repetition was associated with increased gamma power to the preceding stimulus, suggesting a direct relation between increased background high frequency ‘noise' activating extraneous neural circuits during early-stimulus processing that could disrupt refractory-based circuit inhibition to the next repetition of that stimulus. Of note, N1 amplitudes were heterogeneous and suggest a subset of individuals with abnormally high N1, but habituation and gamma function were more consistently abnormal and associated with clinical measures.

Certain limitations in this study need to be considered. First, we cannot determine the degree to which our findings represent FXS-specific factors or alterations associated with developmental or intellectual disabilities. For example, there is evidence that individuals with Down syndrome may also show abnormal habituation to repetitive auditory stimuli,^45, 46^ though individuals with Down syndrome generally show decreased or normal ongoing and evoked high beta and gamma power,^47, 48^ which is opposite of that observed here in FXS. Second, FXS participants were taking various medications, and their potential impact on the data, while not apparent in statistical analyses, cannot be ruled out. Third, clinical correlations were performed on an exploratory basis only, and require validation via replication in additional samples. Last, although no gender differences were found, we had a limited number of FXS female participants.

To the best of our knowledge, this study presents the first comprehensive analysis of short-term auditory habituation in FXS. Our findings are consistent with a model that abnormalities in local circuit function leads to a hyper-excitability of sensory cortex that contributes to altered sensory experiences in FXS and other behavioral problems. Given the proposed linkage of circuit-based abnormalities in FXS with sensory processing abnormalities, reduced habituation and gamma abnormalities may represent promising candidate translational biomarkers for FXS. In FXS mice, minocycline treatment reduces audiogenic seizures,^49^ partially reduces aberrant N1 amplitudes and improves N1 habituation,^18^ suggesting that circuit dynamics associated with habituation may be good translational targets for drug development. Gamma abnormalities in particular may help track and explain hypothesized excitatory/inhibitory imbalances in FXS, and may contributed to lower seizure thresholds. Concurrent work with EEG in FXS mouse models^50^ will further elucidate molecular mechanisms underlying these electrophysiological alterations and their potential use as translational biomarkers for new targeted treatments for FXS. Such work might also lead to new treatment options for neural hyper-excitability and sensory sensitivities in a subgroup of patients with idiopathic autism.

## Figures and Tables

**Table 1:** Participant characteristics

	*FXS*, n=*14*				*Controls*, n=*15*			
	*Mean*	*s.d.*	*Range*		*Mean*	*s.d.*	*Range*	t *statistic (df)*
Age	28.5	11.7	14–57	Age	28.9	10.2	16–55	*t*(27)=0.1, *P*=0.91
Full scale IQ	54.9	16.1	47–94	Full scale IQ	103.9	12.9	82–118	*t*(27)=9.1, *P*<0.001
Verbal	2.7	3.5	1–11	Verbal	103.0	8.7	90–119	
Nonverbal	2.0	2.0	1–7	Performance	110.2	10.4	89–124	
SCQ	21.8	6.7	14–31	SCQ	4.7	4.9	1–17	*t*(20)=6.9, *P*<0.001
Sensory profile	33.1	9.3	19–46	Sensory profile	23.4	4.1	17–30	*t*(21)=3.2, *P*=0.004
ABC irritability	9.0	9.8	0–26					
ABC hyperactivity	9.1	8.8	0–26					
ABCL withdrawn	59.9	13.8	50–93					
ABCL anxiety/depression	58.4	10.8	50–84					

Abbreviations: ABC, aberrant behavior checklist; ABCL, Achenbach adult and child behavior checklists; df, degree of freedom; FXS, Fragile X Syndrome; IQ, intelligence quotient; SCQ, social and communication questionnaire; WASI, Wechsler abbreviated scale of intelligence.

IQ assessed by Stanford Binet in FXS and estimated using the WASI Wechsler scale in healthy controls.

**Table 2:** Time–frequency clusters with significant group differences

*Cluster label*	*Time range*	*Peak time*	*Peak frequency*	*Statistic*	Direction of group difference
*Phase locking (ITC)*					
Initial stimulus pre-N1 delta/theta	−22 to 30 ms	−4 ms	5 Hz	*t*(27)=3.37, *P*=0.002	FXS>CON
Initial stimulus N1 gamma	20 to 210 ms	56 ms	35 Hz	*t*(27)=3.19, *P*=0.004	CON>FXS
Initial stimulus N2 delta/theta	254 to 322 ms	297 ms	5 Hz	*t*(27)=3.51, *P*=0.002	CON>FXS
Repetition 1 N1 alpha/beta	556 to 658 ms	642 ms	13 Hz	*t*(27)=3.09, *P*=0.005	FXS>CON
Repetition 1 N1 gamma	530 to 598 ms	556 ms	36 Hz	*t*(27)=4.05, *P*<0.001	CON>FXS
Repetition 1 N2 delta/theta	796 to 918 ms	849 ms	4 Hz	*t*(27)=2.79, *P*=0.009	CON>FXS
Repetition 2 N1 alpha	1056 to 1186 ms	1134 ms	10 Hz	*t*(27)=3.33, *P*=0.003	FXS>CON
Repetition 2 N2 delta/theta	1350 to 1522 ms	1392 ms	3 Hz	*t*(27)=2.90, *P*=0.007	CON>FXS
Repetition 3 N1 alpha	1660 to 1720 ms	1685 ms	11 Hz	*t*(27)=3.02, *P*=0.005	FXS>CON
Repetition 3 N1 gamma	1574 to 1616 ms	1591 ms	42 Hz	*t*(27)=4.14, *P*<0.001	CON>FXS
Repetition 3 N2 delta/theta	1806 to 2030 ms	1867 ms	5 Hz	*t*(27)=3.91, *P*<0.011	CON>FXS
Repetition 3 N2 gamma	1806 to 1910 ms	1884 ms	49 Hz	*t*(27)=4.36, *P*<0.001	CON>FXS

*Single trial power*					
Overall gamma	−220 to 2350 ms	1798 ms	39 Hz	F(1,27)=10.1, *P*=0.004^a^	FXS>CON

*Baseline-corrected single trial power*					
Initial stimulus N1 gamma	64 to 124 ms	108 ms	50 Hz	*t*(27)=3.21, *P*=0.003	CON>FXS
Repetition 1 N1 gamma	512 to 564 ms	539 ms	48 Hz	*t*(27)=2.42, *P*=0.02	CON>FXS
Repetition 2 N1 delta/theta	1030 to 1176 ms	1073 ms	2 Hz	*t*(27)=4.69, *P*<0.001	FXS>CON
Repetition 2 N1 gamma	934 to 1150 ms	961 ms	37 Hz	*t*(27)=3.27, *P*=0.003	CON>FXS
Repetition 3 N1 alpha/beta	1600 to 1702 ms	1668 ms	13 Hz	*t*(27)=2.93, *P*=0.007	FXS>CON

Abbreviations: ANOVA, analysis of variance; CON, control; ERP, event-related potential; FXS, Fragile X Syndrome; ITC, inter-trial coherence.

Cluster names are identified by stimulus number (Initial stimulus or repetition 1, 2 or 3), ERP associated with the time period (if applicable) and frequency band. Time ranges are given relative to initial stimulus onset, to provide equivalency with the *x*-axes in Figure 2. Repetitions occurred every 500 ms. Cluster peaks are identified for highest *t*-values, not for peaks of activity. For overall gamma single trial power, a peak statistic is reported, however, it should be noted that group differences were remarkably stable throughout the time period.

a Number of trials retained was significantly correlated with overall gamma single trial power in FXS, so number of trials was included as a factor in an ANOVA for this group comparison only. Group differences remained significant.

**Table 3:** Significant clinical correlations

*EEG measure*	*Clinical scale*				
	*Sensory profile—auditory*	*ABCL—withdrawn*	*ABC—hyperactivity*	*ABC—irritability*	*SCQ total score*
N1 amplitude—initial tone	−0.67*	—	—	—	—
Percent change N1 amplitude—asymptote (final repetition)	−0.65*	−0.81**	0.76*	—	—
Percent change N1 ITC—asymptote (final repetition)	0.59*	−0.89**	—	—	—
N2 amplitude	0.74*	—	—	0.92***	0.78*
Delta/theta ITC (N2 initial stimulus)	−0.64*	—	—	—	—
Alpha/beta ITC (N1 first repetition)	0.67*	—	—	0.69*	—
Alpha ITC (N1 second repetition)	0.69*	—	—	—	—
Alpha ITC (N1 final repetition)	0.67*	0.76*	0.77**	0.74*	—
Baseline-corrected gamma STP (N1 initial stimulus)	—	0.67*	0.70*	0.68*	0.89**
Alpha/beta STP (N1 final repetition)	0.61*	—	—	—	—

Abbreviations: ABC, aberrant behavior checklist; ABCL, Achenbach adult and child behavior checklists; df, degree of freedom; EEG, electroencephalography; ITC, inter-trial coherence; NS, not significant; SCQ, social and communication questionnaire.

*Note:* for amplitude correlations on negative ERP components N1 and N2 (first and fourth items in the first column), negative correlations indicate an increased amplitude of response correlated to increased scores on the clinical scale, while positive correlations indicate a decreased amplitude of response correlated to increased scores on the clinical scale. EEG measures with no significant correlations to clinical variables are not included. All correlations are Spearman's *ρ*.

'—' indicates NS.

*P<0.05. **P<0.01. ***P<0.001.
