# Supplementary material for: Reduced habituation of auditory evoked potentials indicate cortical hyper-excitability in Fragile X Syndrome
Source: Transl Psychiatry. 2016 Apr 19;6(4):e787–. doi: 10.1038/tp.2016.48 (PMC4872406; doi:10.1038/tp.2016.48)
Supplement: Supplementary Information [file tp201648x1.docx]

*ERP Spatial PCA.*

For each condition, PCA with promax (oblique) vector rotation and Kaiser normalization^1^ was calculated on a 128X128 sensor covariance matrix (1500 time-points as observations) from the grand average of all subjects. Scree tests^2^ identified 1 component accounting for 93.0 percent of the variance). Component weights was multiplied by each subject’s single trial data, summed across sensors, and divided by the sum of the component weights, reducing waveforms from one for each sensor to one waveform per component for each subject^3^ (Figure 1).

Prior to the final PCA analyses, spatial PCA was conducted using the same procedure described above on the grand averages for each group (FXS and control) separately to verify that component topographies did not differ between groups. Spatial PCA produced 1component for each group, which accounted for 90.0% and 89.7% of the variance for FXS and controls, respectively. Component weights for the two groups were correlated r=.99, p=.001, suggesting no difference between topographic distribution between groups and justifying collapsing over group to produce a final set of PCA weights directly comparable between groups.

References:

1. Dien, J., Khoe, W., & Mangun, G. R. (2007). Evaluation of PCA and ICA of simulated ERPs: Promax vs. Infomax rotations. *Human brain mapping, 28*(8): 742-763.

2. Cattell, R. B. (1966). Evaluating therapy as total personality change: theory and available instruments. *American journal of psychotherapy, 20*(1): 69-88.

3. Spencer, K.M., Dien, J., Donchin, E. (2001). Spatiotemporal analysis of the late ERP responses to deviant stimuli. *Psychophysiology, 38*(2): 343-358.

Supplemental Figure 1.


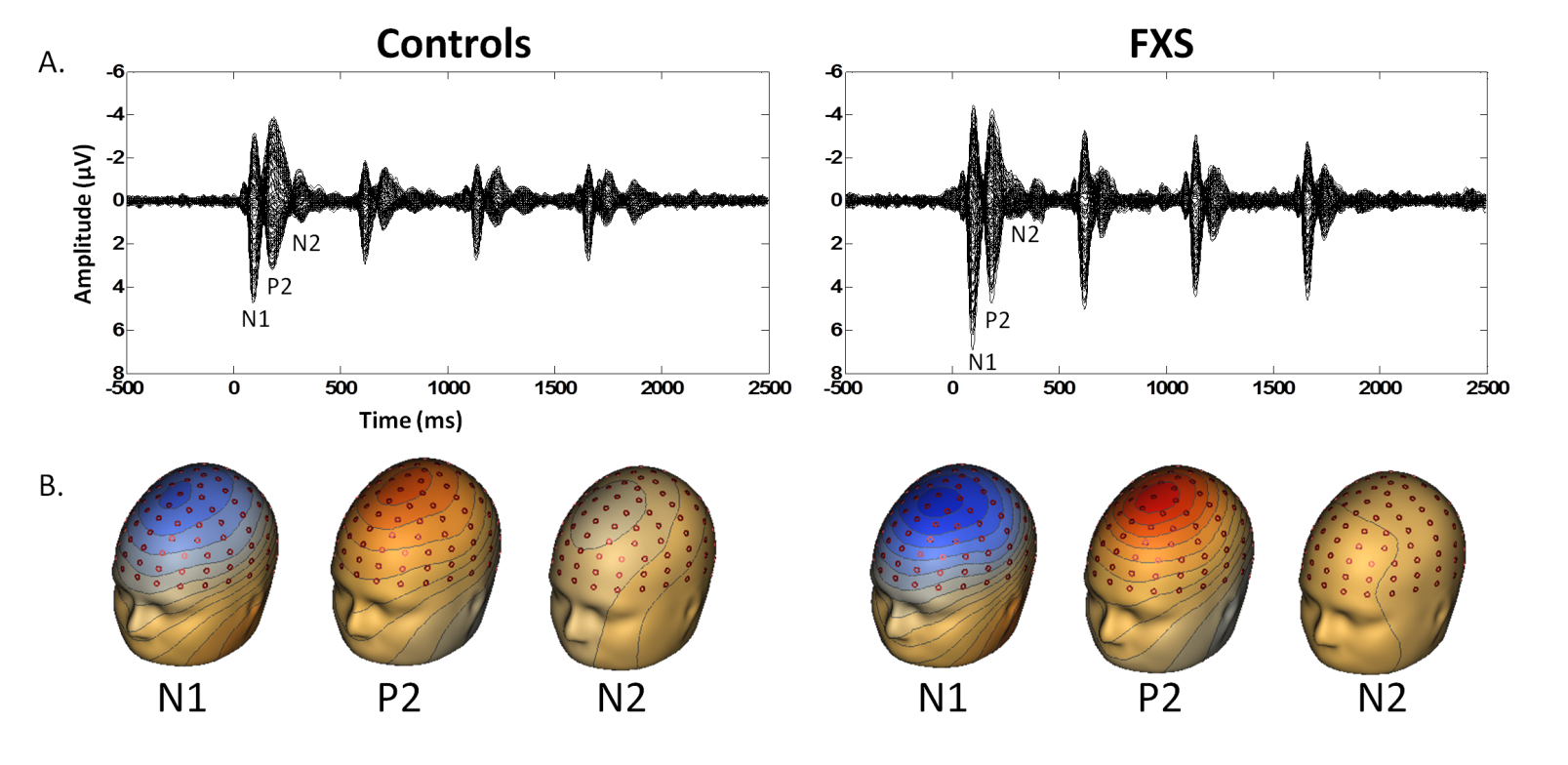


A. Traditional ERP butterfly plots for each group, with N1, P2, and N2 peaks labeled for the initial stimulus.

B. Spatial topographies for the N1, P2, and N2 waveforms to the initial stimulus for each group, showing similar topographies across ERP components and groups, except for N2, which is largely absent or severely delayed in FXS. All topographies are on the same scale.

Supplementary Figure 2.


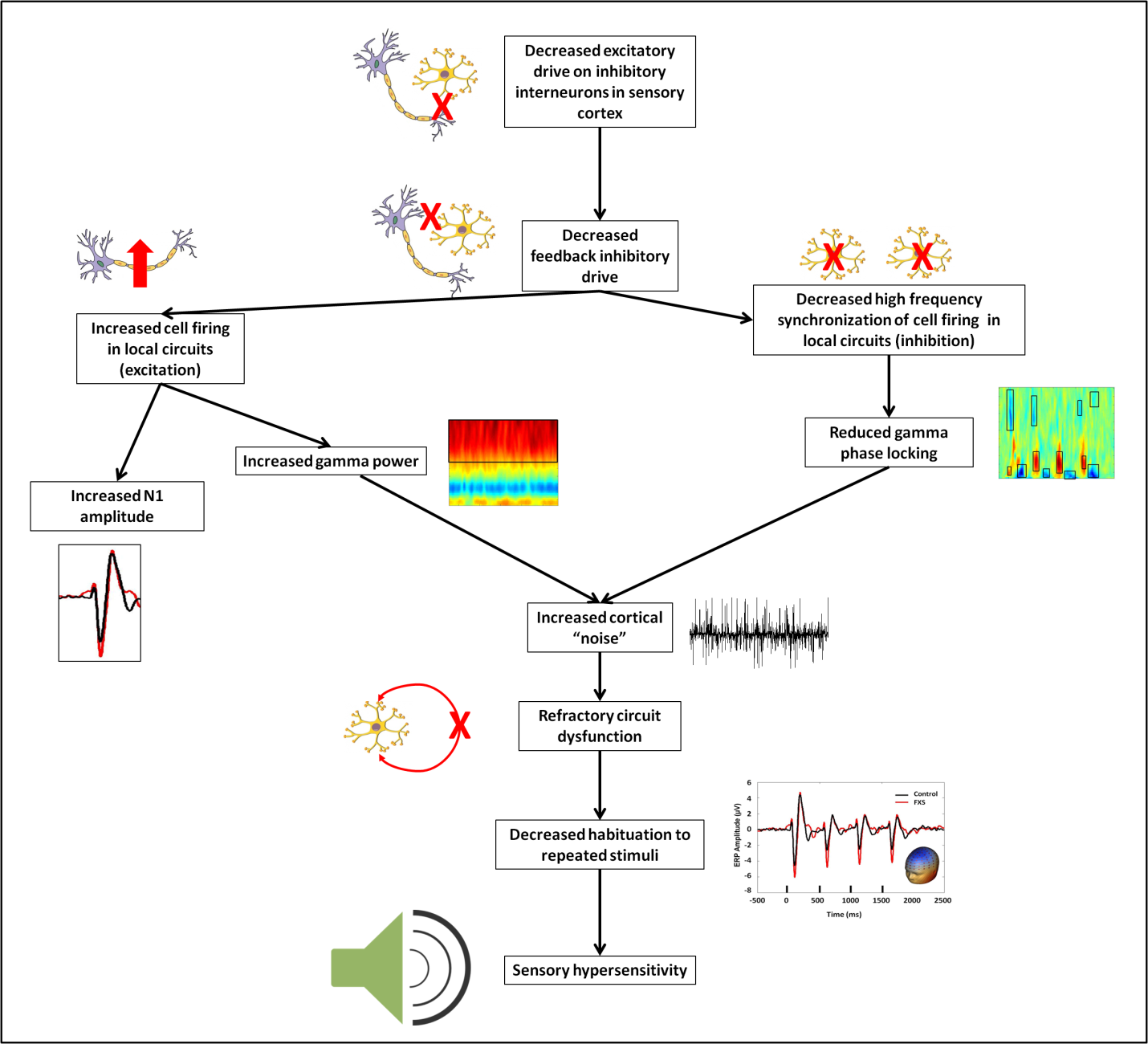


Schematic representation of hypothesized mechanism for contribution of local interneuron circuit dysfunction to reduced habituation and increased sensory hypersensitivity.
